# Avoiding C-hacking when evaluating survival distribution predictions with discrimination measures

**DOI:** 10.1093/bioinformatics/btac451

**Published:** 2022-07-12

**Authors:** Raphael Sonabend, Andreas Bender, Sebastian Vollmer

**Affiliations:** Department of Computer Science, Technische Universität Kaiserslautern, 67663 Kaiserslautern, Germany; Engineering Department, University of Cambridge, CB2 1PZ Cambridge, UK; MRC Centre for Global Infectious Disease Analysis, Jameel Institute, Imperial College London, School of Public Health, W2 1PG London, UK; Department of Statistics, LMU Munich, 80539 Bavaria, Germany; Department of Computer Science, Technische Universität Kaiserslautern, 67663 Kaiserslautern, Germany; Data Science and its Application, Deutsches Forschungszentrum für Künstliche Intelligenz (DFKI), 67663 Kaiserslautern, Germany; Mathematics Institute, University of Warwick, CV4 7AL Coventry, UK

## Abstract

**Motivation:**

In this article, we consider how to evaluate survival distribution predictions with measures of discrimination. This is non-trivial as discrimination measures are the most commonly used in survival analysis and yet there is no clear method to derive a risk prediction from a distribution prediction. We survey methods proposed in literature and software and consider their respective advantages and disadvantages.

**Results:**

Whilst distributions are frequently evaluated by discrimination measures, we find that the method for doing so is rarely described in the literature and often leads to unfair comparisons or ‘C-hacking’. We demonstrate by example how simple it can be to manipulate results and use this to argue for better reporting guidelines and transparency in the literature. We recommend that machine learning survival analysis software implements clear transformations between distribution and risk predictions in order to allow more transparent and accessible model evaluation.

**Availability and implementation:**

The code used in the final experiment is available at https://github.com/RaphaelS1/distribution_discrimination.

## 1 Introduction

Predictive survival models estimate the distribution of the time until an event of interest takes place. This prediction may be presented in one of three ways, as a: (i) time-to-event, $Y\in {\mathbb{R}}_{>0}$, which represents the time until the event takes place; (ii) a relative risk, ϕ∈ℝ, which represents the risk of the event taking place compared to other subjects in the same sample; or (iii) the probability distribution for the time to the event, $S\in \text{Distr}({\mathbb{R}}_{>0})$, where $\text{Distr}({\mathbb{R}}_{>0})$ is the set of distributions over ${\mathbb{R}}_{>0}$. Less abstractly, consider the Cox Proportional Hazards (CPH) model (Cox, 1972): $h(t)=h_{0}(t)\;\text{exp}\;(X\beta )$ where *h*_0_ is the ‘baseline’ hazard function, *X* are covariates, and *β* are coefficients to be estimated. In practice, software fits the model by estimating the coefficients, $\hat{\beta }$. Predictions from the fitted model may then be returned as either a relative risk prediction, $X\hat{\beta }$ or $\text{exp}\;(X\hat{\beta })$, or *h*_0_ is also estimated and a survival distribution is predicted as $\hat{h}(t)={\hat{h}}_{0}(t)\;\text{exp}\;(X\hat{\beta })$.

The CPH is a special type of survival model that can naturally return both a survival distribution and a relative risk prediction, however, this is not the case for all models. For example, random survival forests (RSFs) (Ishwaran *et al.*, 2008) only return distribution predictions by recursively splitting observations into increasingly homogeneous groups and then fitting the Nelson–Aalen estimator in the terminal node.

The most common method of evaluating survival models is with discrimination measures (Collins *et al.*, 2014; Gönen and Heller, 2005; Rahman *et al.*, 2017), in particular Harrell’s (Harrell *et al.*, 1982) and Uno’s C (Uno *et al.*, 2011). These measures determine if relative risk predictions are concordant with the true event time. To give a real-world example, a physician may predict that a 70-year-old patient with cancer is at higher risk of death than a 12-year-old patient with a broken arm. If the 70-year-old dies before the 12-year-old then the risk prediction is said to be concordant with the observed event times as the patient with the predicted higher risk died first.

Despite discrimination measures being so common, it transpires that they are very easy to manipulate. In this article, we will define ‘C-hacking’, discuss how it can occur, and how to avoid it. We will focus on models that make survival distribution predictions as these are the primary source of accidental C-hacking. Note we are concerned only with how discrimination measures are utilized for model comparison and not about the properties of the measures themselves. For example, we are interested in *how* to transparently compare if an RSF (native distribution prediction) has better discrimination than a support vector machine (native risk prediction only) (Van Belle *et al.*, 2011); but we are not interested in *which* measure to use. By ‘native’ prediction, we mean the prediction that is made by a model after fitting without further transformations or post-processing.

First, we define C-hacking, before reviewing methods of how to evaluate distribution predictions with measures of discrimination and discussing their advantages and disadvantages. We do not consider the competing risks setting, which requires specialized measures.

## 2 C-hacking

We define ‘C-hacking’ broadly as an inappropriate comparison of survival models with measures of concordance that can occur accidentally or deliberately. We have identified three primary types of C-hacking: (I) evaluating models with multiple concordance indices and only reporting the index that is most beneficial to the authors; (II) reporting multiple different types of concordance indices as one generic ‘c-index’; and (III) evaluating the discriminatory ability of models that make survival distribution predictions without clearly justifying prediction transformations and/or measure choices.

Our motivating example in Section 4 demonstrates how simple it is for the first two forms of C-hacking to occur. In that example, the hypothetical authors of the experiment could state that their CPH model outperforms the RSF by selecting one measure (Type I C-hacking) after viewing all results (‘according to Antolini’s C, the CPH outperforms the RSF’), or they could state the RSF outperforms the CPH by erroneously conflating (Type II C-hacking) two different concordance indices (‘the RSF outperforms the CPH with a C-index of 0.897 compared to 0.852’).

Avoiding Types I and II C-hacking depend on the same protocol as avoiding p-hacking (Head *et al.*, 2015), i.e. planning the evaluation protocol in advance including selecting the chosen discrimination measure (or measures), and ensuring all calculated results are clearly reported. Journals should be aware of C-hacking and should insist on clear reporting of discrimination measures to avoid it.

In contrast, Type III C-hacking is more complex and as such is more likely to occur accidentally and requires expert knowledge to be avoided. It can also occur in different contexts. For example, papers that compare models with different prediction types may be C-hacking by omitting the transformation used to evaluate distribution predictions with time-independent (Section 3.4) discrimination measures (e.g. Fernández *et al.*, 2016; Herrmann *et al.*, 2021; Spooner *et al.*, 2020; Zhang *et al.*, 2021)—this is C-hacking as the native prediction is not being evaluated but instead an unknown pipeline and therefore it can greatly mislead about general model performance. In another example, one may erroneously compare the discrimination of a distribution-predicting model with Antolini’s C (Antolini *et al.*, 2005), to the discrimination of a risk predicting model with Harrell’s C—this would be C-hacking as two different mathematical objects are being directly compared with two different measures (thus any comparison is virtually meaningless). Note: *separately* reporting the discrimination from distribution-predictions and risk predictions is valid as these are different prediction types, it is only ‘hacking’ if they are treated as the same or used to generalize about model performance.

## 3 Materials and methods

We consider how discrimination measures are utilized in the literature to evaluate the predictive performance of models that predict survival distributions (Section 3.1), we then review the identified methods (Sections 3.3 and 3.4). To illustrate our findings, we provide a worked example in Section 4. The focus in our review is not to compare the (dis)advantages of measures but instead their compatibility. For example, we do not compare if Antolini’s C is ‘better’ than Harrell’s C but instead note that the former evaluates distribution predictions and the latter risk predictions.

### 3.1 Literature review

We first performed a formal literature review using PubMed and then a less formal review from articles and software packages that had been drawn to our attention. The purpose of the review was to determine how model discrimination predictions have historically been evaluated for machine learning models that make distributional predictions.

**Fig. 1.**
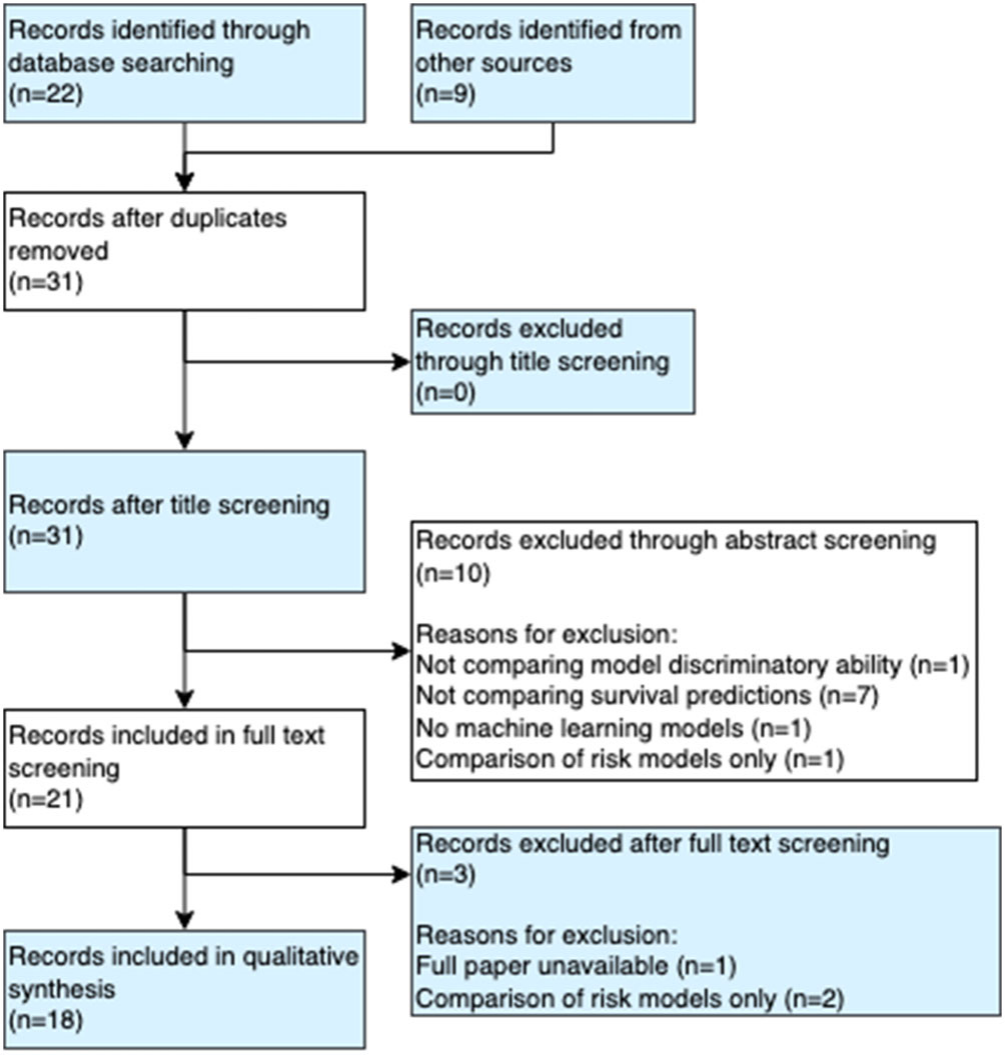
PRISMA diagram for literature review. Database: PubMed. Search terms: ‘(comparison OR benchmark) AND (“survival analysis” OR “time-to-event analysis”) AND “machine learning” AND (discrimination OR concordance OR “C statistic” OR “c index”)’. Inclusion criteria: articles that compare machine learning survival predictions with measures of discrimination

We searched PubMed for ‘(comparison OR benchmark) AND (“survival analysis” OR “time-to-event analysis”) AND “machine learning” AND (discrimination OR concordance OR “C statistic” OR “c index”)’. We excluded articles if: (i) they did not use measures of discrimination; (ii) no machine learning models were included; (iii) only risk-prediction models were included; and (iv) the models did not make survival predictions (e.g. classifiers). We found 22 articles in our initial search, which were reduced to nine after screening, a full PRISMA diagram is provided in Figure 1; the diagram includes nine other records which were identified outside of the search and which are also discussed below.

We retained nine articles from our PubMed search for qualitative synthesis: Hadanny *et al.* (2022), Johri *et al.* (2021), Loureiro *et al.* (2021), Mosquera Orgueira *et al.* (2020), Aivaliotis *et al.* (2021), Kantidakis *et al.* (2020), Spooner *et al.* (2020), Crombé *et al.* (2021) and Herrmann *et al.* (2021). All of these, without exception, compared risk-predicting Cox-based models (e.g. regularized, boosted, neural adaptations) to RSFs Ishwaran *et al.* (2008). **scikit-survival** (Pölsterl, 2020), **randomForestSRC** (Ishwaran and Kogalur, 2022), **ranger** (Wright and Ziegler, 2017) and **mlr** (Bischl *et al.*, 2016) were utilized to implement and evaluate the RSFs. RSFs make distributional predictions by ensembling a Nelson–Aalen estimator across bootstrapped models (Ishwaran *et al.,* 2008). Transformation from distribution to risk is handled in **randomForestSRC** and **scikit-survival** by taking the sum over the predicted cumulative hazard function for each observation, which is recommended by Ishwaran *et al.* (2008), we refer to this transformation as ‘expected mortality’ (Section 3.4.3). In contrast, no transformation is provided in **ranger**, which only returns a distribution prediction, however, this is handled in Spooner *et al.* (2020) by utilizing **mlr**, which provides the same expected mortality transformation.

Apart from the articles identified from the aforementioned literature review, we were also aware of the following nine articles and software that discuss the discrimination of models that make distributional predictions: Kvamme *et al.* (2019), Lee *et al.* (2018), Gensheimer and Narasimhan (2019), Kvamme and Borgan (2021), Sonabend *et al.* (2021), Zhao and Feng (2020), Haider *et al.* (2020), Mogensen *et al.* (2012) and Schwarzer *et al.* (2000). Of these articles, the methods of comparing predicted distribution discriminatory ability are: (i) utilizing time-dependent concordance indices (Kvamme *et al.*, 2019; Kvamme and Borgan, 2021; Lee *et al.*, 2018) (Section 3.3); (ii) comparing predicted probabilities at a given time-point (Gensheimer and Narasimhan, 2019; Mogensen *et al.*, 2012; Schwarzer *et al.*, 2000; Zhao and Feng, 2020; Zhong and Tibshirani, 2019) (Section 3.4.1); and (iii) calculating and comparing a summary statistic (e.g. expected survival time) from the predicted distributions (Haider *et al.*, 2020; Sonabend *et al.*, 2021) (Section 3.4.2).

We discuss the methods listed above (expected mortality, time-dependent concordance indices, comparing predicted probabilities, and comparing summary statistics) in two groups: (A) time-dependent discrimination measures; and (B) time-independent discrimination measures. Discussion follows after defining notation.

### 3.2 Notation

Throughout the article, we use the following notation: let $X_{i}\in {\mathbb{R}}^{p}$ be *p* covariates for subject *i*, let *Y_i_* be the true (but unobserved) survival time; *C_i_* be the true (but unobserved) censoring time, and $T_{i}=\min(Y_{i},C_{i})$ be the observed outcome time; finally, let $\Delta_{i}=I(T_{i}=Y_{i})$ be the survival indicator.

In practice, software for time-to-event predictions will usually return a matrix of survival probabilities. Let $\left[T_{0},T_{N}\right]$ be the range of observed survival times in a training dataset, let *M* be the number of observations in the test dataset and let *K* be the number of time-points for which predictions are made, then we predict $S\in {\left[0,1\right]}^{M\times K}$, which correspond to predictions of individual survival functions, $S_{i}(T),T\in T\subseteq [T_{0},T_{N}]$.

### 3.3 Time-dependent discrimination

Discrimination measures can be computed as the proportion of concordant pairs over comparable pairs. Let i≠j be a pair of observations with observed outcomes and predicted risks of $\left\{(T_{i},\Delta_{i},\phi_{i}),(T_{j},\Delta_{j},\phi_{j})\right\}$ respectively. Then (*i*, *j*) are comparable if $\left(T_{i}<T_{j})\cap (\Delta_{i}=1\right)$ and the predicted risks are concordant with the outcome times if $\phi_{i}>\phi_{j}$. In this article, we are concerned with how the values of $\left(\phi_{i},\phi_{j}\right)$ are calculated (from distributional predictions).

Time-dependent discrimination measures define concordance over time either by taking $\phi_{i}$ to be predicted survival probabilities such as Antolini *et al.* (2005), or as predicted linear predictors, such as Heagerty *et al.* (2000).

Antolini *et al.* (2005) define a pair of observations as concordant if the predicted survival probabilities are concordant at the shorter outcome time,

(1) $$P({\hat{S}}_{i}(T_{i})<{\hat{S}}_{j}(T_{i})|T_{i}<T_{j}\cap \Delta_{i}=1)$$

In contrast, Heagerty *et al.* (2000) and Heagerty and Zheng (2005) calculate the Area Under the Curve (AUC) by integrating over specificity and sensitivity measures given by

(2) $${\text{TPR}}_{t}(c)=P(\phi_{i}>c|T_{i}\leq t)$$

 

(3) $${\text{TNR}}_{t}(c)=P(\phi_{i}\leq c|T_{i}>t)$$

 

(4) $${\text{ROC}}_{t}(p)={\text{TPR}}_{t}\{{\left[1-{\text{TNR}}_{t}(p)\right]}^{-1}\}$$

 

(5) $$\text{AUC}(t)=\int_{0}^{1}{\text{ROC}}_{t}(p)\;dp$$

where *c* is a threshold for the predicted risk and *t* is a cutoff value for the survival time. These values can be interpreted similarly to the classification setting where a true positive is correctly predicting that an event occurs before time *t*, where a prediction of the event is defined by a relative risk greater than some threshold, $\phi_{i}>c|T_{i}\leq t$. Whereas a true negative is correctly predicting that an event does not occur (predicted risk less than the threshold) before the given time, $\phi_{i}\leq c|T_{i}>t$. Weighting the final AUC equation provides an estimate of concordance, $P(\phi_{i}>\phi_{j}|T_{i}<T_{j})$, via well-established results (Agresti, 2002; Harrell *et al.*, 1996; Heagerty and Zheng, 2005; Korn and Simon, 1990). Various metrics have been based on Heagerty’s equations and several are implemented in the R package **survAUC** (Potapov *et al.*, 2012). However, all require a single relative risk predictor, and therefore require some transformation from a survival distribution prediction, and secondly all assume a one-to-one relationship between the predicted value and expected survival times (which is unlikely in complex machine learning models), for example a proportional hazards assumption where the predicted risk is related to the predicted survival distribution by multiplication of a constant (Potapov *et al.*, 2012).

We are unaware of any time-dependent AUC metrics, except for Antolini’s, that evaluates survival time predictions without a further transformation being required. This may explain why Antolini’s C-index is seemingly more popular in the artificial network survival literature (Kvamme *et al.*, 2019; Kvamme and Borgan, 2021; Lee *et al.*, 2018).

On the surface, time-dependent discrimination measures are optimal for evaluating distributions by discrimination. However, they are difficult to use for model comparison or tuning because different models can be superior at different time points. Time-dependent measures that evaluate risk predictions (such as Heagerty’s) require a transformation from survival distribution predictions and any such transformation is unlikely to result in the one-to-one mapping required by the measures. In contrast, Antolini’s C evaluates the concordance of a distribution, which means that it can only be used to compare the concordance of two models that make distribution predictions, as opposed to, say, one model that predicts distributions (e.g. RSFs) and one that predicts relative risks (e.g. Support Vector Machine (SVMs)). The experiment in Section 4 demonstrates why results from Antolini’s C cannot be simply compared to results from other concordance indices.

### 3.4 Time-independent discrimination

Time-independent discrimination measures for survival analysis evaluate relative risk predictions by estimating concordance.

Let $S\subseteq \text{Distr}({\mathbb{R}}_{>0})$ be a convex set of distributions over the positive Reals; then we define a *distribution reduction method* as any function of the form: f:S→ℝ, which maps a survival distribution prediction, ζ∈S, to a single relative risk, ϕ∈ℝ. In practice, we consider the discrete analog and functions $f\prime :{\left[0,1\right]}^{K}\to \mathbb{R}$.

Distribution reduction methods are required to utilize time-independent discrimination measures for models that make distribution predictions. We consider the three from the literature review in turn.

#### 3.4.1 Comparing probabilities

Evaluating discrimination at a given survival time is formally defined by estimating

(6) $$P({\hat{S}}_{i}(t)<{\hat{S}}_{j}(t)|T_{i}<T_{j}\cap \Delta_{i}=1)$$

for some chosen $t\in {\mathbb{R}}_{>0}$. The distribution is reduced to a relative risk by evaluating the survival probabilities at a given time-point, $\phi :=\hat{S}(t\prime )$ where $\hat{S}$ is the predicted survival function and $t\prime \in {\mathbb{R}}_{>0}$. Note the key difference between this method and Antolini’s C is that *t* can be arbitrarily chosen here, whereas Antolini’s C estimates the concordance at the observed outcome times.

This method assesses how well a model separates patients at a single time-point; it has several problems: (i) it is not ‘proper’ in the sense that the optimal model may not maximize the concordance at t′ (Blanche *et al.*, 2019); (ii) it is prone to manipulation as one could select the t′ that maximizes the C-index for their chosen model (see Section 4); and (iii) if predicted survival curves overlap then evaluation at different time-points will lead to contradictory results (as the observed event times will always stay the same). The above issues apply even if evaluated at several time-points.

#### 3.4.2 Distribution summary

The distribution summary statistic method reduces a probability distribution prediction to a summary statistic, most commonly, the mean or median of the distribution, i.e.

(7) $$P(E[\zeta_{i}]<E[\zeta_{j}]|T_{i}<T_{j}\cap \Delta_{i}=1)$$

 

(8) $$P(m(\zeta_{i})<m(\zeta_{j})|T_{i}<T_{j}\cap \Delta_{i}=1)$$

where $m(\zeta_{i})$ is the median of distribution *ζ_i_*. In theory, this should provide the most meaningful reduction with a natural interpretation (mean or median survival time), however, this is not the case as the presence of censoring means that the predicted survival predictions will usually result in ‘improper predictions’, i.e. the basic properties of the survival function are not satisfied: $\underset{t\to +\infty }{\lim}S_{T}(t)\neq 0$. To see why this is the case, note that the majority of survival distribution predictions make use of a discrete estimator such as the Kaplan–Meier estimator, which is defined as follows:

(9) $$\hat{S}(t)=\{\begin{array}{cc} 1 & t<t_{\left(1\right)}\\ \prod_{i:t_{\left(i\right)}\leq t}\left(1-d_{i}/n_{i}\right) & t\geq t_{\left(1\right)} \end{array}$$

where *d_i_*, *n_i_* are the number of deaths and events (death or censoring) at ordered events times time $t_{\left(i\right)},i=1,\ldots ,n$. By definition of this estimator, unless all observations at risk in the final time-point experience the event (*d_i_* = *n_i_*), the predicted survival probability in this last point will be non-zero.

**Fig. 2.**
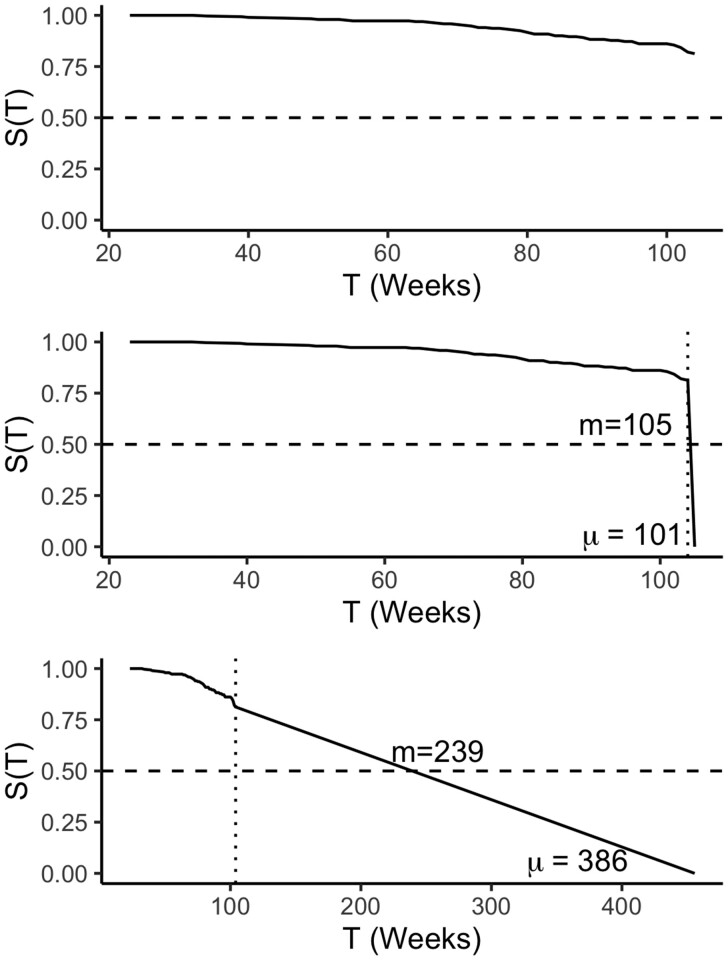
Extrapolation methods to ‘fix’ improper distribution predictions. Top: Kaplan–Meier estimator fit on the rats (Mantel *et al.*, 1977) dataset (Table 1), which results in an improper distribution as $\underset{T\to \infty }{\lim}=0.81\neq 0$. Middle: Dropping the survival probability to zero at *T *=* *105, just after the study end. Bottom: Dropping the survival probability to zero by linearly extrapolating from first, (S(T)=1,T=0), and last, (S(T)=0.81,T=104), observed survival times. Dashed horizontal lines are drawn at S(T)=0.5 and dotted vertical lines at *T *=* *104, where the observed data ends and the extrapolation begins. Median (*m*) and mean (*μ*) are provided for both extrapolation methods. Both methods result in quantities skewed heavily toward the final extrapolated time. For the ‘dropping’ method the median is exactly at the final time. Linear extrapolation results in probabilities that are unrealistically large (a lab rat lives 2 years on average)

Several methods have been considered to extrapolate predictions to fix this problem, such as dropping the last predicted probability to zero either at or just after the last observed time-point (Sonabend *et al.*, 2021), or by linear extrapolation from the observed range (Haider *et al.*, 2020) (Fig. 2). However, these methods require unjustifiable assumptions and result in misleading quantities. For example, dropping the survival probability to zero immediately after the study end assumes that all patients (no matter their risk) instantaneously die at the same time, which will skew the distribution mean and median toward the final event time (Haider *et al.*, 2020). The extrapolation method has the opposite problem, if the prediction survival curves are shallow then the extrapolated predictions can easily result in impossible (or at least highly unrealistic) values (Fig. 2).

However, we note that summarizing a ‘proper’ distribution prediction (i.e. one that doesn’t violate the limit properties) by its mean or median will provide a natural relative risk. But this is rarely the case for all predicted distributions in a test set and so the problem remains.

#### 3.4.3 Expected mortality

The final time-independent discrimination method estimates

(10) $$P(\phi_{i}>\phi_{j}|T_{i}<T_{j}\cap \Delta_{i}=1)$$

where

(11) $$\phi_{i}:=\sum_{t\in T}-\text{log}\;{\hat{S}}_{i}(t)=\sum_{t\in T}{\hat{H}}_{i}(t)$$

and ${\hat{H}}_{i},{\hat{S}}_{i}$ are the predicted cumulative hazard and survival functions respectively. Summing over the predicted cumulative hazard provides a measure of expected mortality for similar individuals (Hosmer *et al.*, 2011; Ishwaran *et al.*, 2008) and a closely related quantity can even be used as measure of calibration (Van Houwelingen, 2000).

The advantage of this method is that it requires no model assumptions, nor assumptions about the survival distribution before or after the observed time period, and finally, the reduction method provides an interpretable quantity that is meaningful as a relative risk: the higher the expected mortality, the greater the risk of the event.

## 4 Motivating example

We now present a motivating example to make clear why these different concordance measures cannot be directly compared in model evaluation and why it is important to be precise about which method is utilized in model comparison studies.

***Experiment design***. We split the rats dataset (Table 1) from R package **survival** (Therneau, 2022) into a random holdout split with two-thirds of the dataset for training and one-third for testing; a seed was set for reproducibility. With the training data we fit a CPH with package **survival**, RSF with package **ranger** and gradient boosting machine with C-index optimization (GBM) (Mayr and Schmid, 2014) with package **mboost** (Hothorn *et al.*, 2020). Note that **ranger** only returns distribution predictions for RSFs and **mboost** only returns risk predictions.

**Table 1.** First five rows of the rats dataset from R package **survival** (Therneau, 2022)

id	Litter	rx	Sex	Time	Status
1	1	1	f	101	0
2	1	0	f	49	1
3	1	0	f	104	0
4	2	1	m	91	0
5	2	0	m	104	0

*Note*: The dataset includes 300 rows, three predictors and the survival outcome as time and status columns.

***Evaluation measures.*** We used each model to make predictions on the holdout data. For the CPH, we made linear predictor predictions with survival::coxph and additionally distribution predictions with survival::survfit. We evaluate the discrimination of all possible predictions with: Harrell’s C, *C_H_*, (Harrell *et al.*, 1982) (‘Harrell’) on the native risk prediction (i.e. returned by package without further user transformation), Uno’s C (Uno *et al.*, 2011) (‘Uno’) on the native risk prediction, Antolini’s C (Antolini *et al.*, 2005) (‘Antolini’), *C_H_* computed on the survival probabilities at every predicted time-point, *C_H_* computed on the distribution mean without any extrapolation (‘Summary (naive)’), *C_H_* computed on the distribution mean with extrapolation method of dropping to zero just after the final time point (‘Summary (extr)’) and *C_H_* computed on the expected mortality (‘ExpMort’). For reporting the concordance computed on survival probabilities at each time-point, we reported the time-point which resulted in the maximum *C_H_* for the RSF, the time-point that resulted in the minimum *C_H_* for the RSF, and one randomly sampled time-point. Note that for the GBM, only *C_H_* and *C_U_* can be computed without a further transformation as GBM’s return risk predictions only. We could have applied a distribution transformation however we could find no examples in the literature where risk predictions are transformed to distributions to then be evaluated by discrimination.

***Results*.** The results (Table 2) indicate how ranking the performance of different algorithms changes depending on the C-index used. The following are examples for how the results in the table could be reported (from most transparent to least):

**Table 2.** Various C-index calculations from different methods and models

Measure	Type	Trafo.	CPH (R)	RSF (D)	GBM (R)
*C_H_*	TI	—	0.859	—	0.831
*C_U_*	TI	—	**0.861**	—	**0.853**
*C_A_*	TD	—	0.852	0.757	—
*C_H_*	TI	Prob (min)	0.500	0.500	—
*C_H_*	TI	Prob (max)	0.859	**0.897**	—
*C_H_*	TI	Prob (rand)	0.859	0.851	—
*C_H_*	TI	Summary (naive)	0.141	0.104	—
*C_H_*	TI	Summary (extr)	0.859	0.871	—
*C_H_*	TI	ExpMort	0.859	0.878	—

*Note*: Included models are Cox PH (CPH), random survival forest (RSF) and gradient boosting machine with C-index optimization (GBM). CPH predicts a risk natively (R) and uses a distribution transformation with a PH model form and Breslow estimator to predict a distribution. RSF predicts a distribution natively (D) and uses an ensemble mortality transformation to predict risk. GBM predicts a risk natively (R). Models are evaluated either with Harrell’s C (*C_H_*), Uno’s C (*C_U_*) or Antolini’s C (*C_A_*). The second column states if a measure is time-independent (TI) or time-dependent (TD). The third column states the transformation required to evaluate a survival distribution prediction with a measure of discrimination, these are: computing *C_H_* on the predicted survival probability at the time-point that results in the smallest value for RSF (‘Prob (min)’); *C_H_* computed on the predicted survival probability at the time-point that results in the largest value for RSF (‘Prob (max)’); *C_H_* computed on the predicted survival probability at an arbitrary time-point (‘Prob (rand)’); *C_H_* computed on the distribution expectation without any extrapolation (‘Summary (naive)’); *C_H_* computed on the distribution expectation after extrapolating by dropping survival probabilities to zero (Fig 2 middle) (‘Summary (extr)’); *C_H_* computed on the expected mortality (‘ExpMort’). Dashes (‘–’) in the final two columns indicate that the given measure is incompatible with the prediction type without transformation. Values in bold are the maximum C-index for that model.

1. CPH is the best performing for distribution predictions under Antolini’s C with a C-index of 0.852 compared to RSF’s 0.757.
2. RSF is the best performing for distribution predictions under the expected mortality transformation with Harrell’s C with a C-index of 0.878 compared to CPH’s 0.859.
3. CPH is the best performing for risk predictions under Uno’s C with a C-index of 0.861 compared to GBM’s 0.853.
4. RSF is the best performing model with a C-index of 0.897, then CPH with C-index of 0.861 and then GBM with C-index of 0.853.

The first three of these are the clearest as they demonstrate what is being evaluated and how. However, the difference between the first two demonstrates how the result can be chosen by the researcher by selecting one measure over another. The final is clearly the least transparent as it mixes many types of predicted types and evaluation measures to draw conclusions.

***Discussion.*** These examples demonstrate how simply reporting ‘the C-index’ without being more precise can lead to manipulation of results (deliberate or otherwise). For example, the absurdly low values for ‘Summary (naive)’ are a result of attempting to calculate the distribution mean from improper distribution predictions, which is easily possible with **lifelines** (Davidson-Pilon, 2019) and **mlr3proba** (Sonabend *et al.*, 2021) (the latter has since been updated in light of this problem). Similarly, despite providing a warning in documentation and on usage, **pec** (Mogensen *et al.*, 2012) still allows concordance evaluation at arbitrary survival points, which could lead to authors reporting the maximum C-index over all time-points (‘Prob (max)’ in Table 2).

It is clear that a shift in reporting is required. When a range of C-indices are tabulated as in Table 2 then dishonest reporting (like the final example above) is clear however in practice a range of values is not reported and instead just a vague ‘C-index’. This problem is analogous to any statistical manipulation, for example p-hacking (Head *et al.*, 2015). The methods of dealing with the problem, ‘C-hacking’, are therefore also the same: researchers should clearly decide at the beginning of an experiment (before running any analyses) what method they will use for evaluating discrimination and state this clearly.

## 5 Conclusions

In this article, we introduced the concept of C-hacking and investigated how this applies to evaluating survival distribution predictions. We reviewed the literature for different methods of evaluating survival distribution predictions with methods of concordance. For time-dependent measures, only Antolini’s C can be directly applied to distribution predictions. This measure can be utilized to compare the discrimination of multiple models that make distribution predictions however as it cannot be applied to models that make risk predictions, its use in benchmark experiments is more limited. In contrast, methods that reduce a distribution prediction to a risk prediction allow for time-independent discrimination measures to be utilized for any combination of survival models. Of the reviewed ‘distribution reduction’ methods that we found in the literature, the expected mortality method of summing over the cumulative hazard was the most robust as it requires no assumptions about the model or prediction and is therefore applicable to all distribution predictions. Once the distribution is reduced to a risk, any time-independent discrimination measure can be applied (e.g. Harrell’s C).

Our motivating example demonstrates why understanding the differences between these methods is so important and how an imprecise statement of methods can lead to manipulation of results. Journals should require clear reporting on how c-statistics are computed in survival analysis to ensure fair reporting of results and to avoid ‘C-hacking’. Furthermore, all open-source software should provide methods to transform distribution to risk predictions, such as the compositions in Sonabend *et al.* (2021).

How to choose and compare these metrics and methods is beyond the scope of this article, however, a simple protocol for evaluating discrimination based on the results above is as follows: (i) select models to compare; (ii) if all models make distribution-predictions then select a time-dependent C-index (e.g. Antolini’s C) otherwise choose a time-independent measure (e.g. Uno’s C); (iii) if there is a combination of risk- and distribution-predicting models then choose a transformation method for analysis (e.g. expected mortality); and (iv) run experiment and report results. Any analysis of discrimination from distribution-predicting models should also be augmented with calibration measures [e.g. D-Calib (Haider *et al.*, 2020)] and proper scoring rules [e.g. Right-censored logloss (RCLL) (Avati *et al.*, 2018)]; formal statistical comparisons such as confidence intervals and/or hypothesis test results should be reported when possible. Whichever metrics are chosen, researchers should be precise about exactly which estimators are utilized and any post-processing of results that was required.

### Author contributions

R.S. conceptualized the article. All authors contributed equally to writing and editing.

## Funding

A.B. has been funded by the German Federal Ministry of Education and Research (BMBF) under grant no. 01IS18036A. The authors of this work take full responsibilities for its content.

*Conflict of Interest*: none declared.
